# Supplementary material for: Membrane cholesterol regulates inhibition and substrate transport by the glycine transporter, GlyT2
Source: Life Sci Alliance. 2023 Jan 23;6(4):e202201708. doi: 10.26508/lsa.202201708 (PMC9873984; doi:10.26508/lsa.202201708)
Supplement: Supplementary file 1 [file LSA-2022-01708_TableS1.docx]

**Table S1 - Thickness (Å) and area per lipid (Å^2^) for the lipid bilayer for 10 μs coarse grain simulations on GlyT2 in a POPC/CHOL membrane with no lipid inhibitors (control) or 20 of the specified lipid inhibitors present.**

| Lipid Inhibitor | Area Per Lipid^a^ | Membrane Thickness^b^ |
| --- | --- | --- |
| None | 58.28±0.41 | 41.35±0.21 |
| OLLys | 58.13±0.68 | 41.33±0.20 |
| OLLeu | 57.94±0.55 | 41.36±0.22 |
| OLTrp | 58.55±0.40 | 41.39±0.20 |
| OLSer | 58.23±0.84 | 41.33±0.21 |
| ^a^Area per lipid is measured for the POPC lipid headgroups | | |

^b^Membrane thickness is measured at the distance between P atoms in the POPC headgroups
