# Supplementary material for: Membrane cholesterol regulates inhibition and substrate transport by the glycine transporter, GlyT2
Source: Life Sci Alliance. 2023 Jan 23;6(4):e202201708. doi: 10.26508/lsa.202201708 (PMC9873984; doi:10.26508/lsa.202201708)
Supplement: Supplementary file 2 [file LSA-2022-01708_TableS2.docx]

**Table S2 - Percentage of the total CG simulation time in which residues are in contact with the OLLeu lipid inhibitors that is bound in the extracellular allosteric pocket of GlyT2.**

Only interactions that occur for >15% of the total simulation time are reported.^a^

| Region | Residue | Occupancy |
| --- | --- | --- |
| TM1 | W215 | 72.5 |
| TM1 | P218 | 48.9 |
| TM1 | Y219 | 46.2 |
| TM5 | L437 | 21.3 |
| TM5 | G440 | 18.1 |
| TM5 | V441 | 34.1 |
| TM7 | V751 | 51.7 |
| TM7 | I520 | 52.8 |
| TM7 | I524 | 86.8 |
| TM7 | F526 | 16.8 |
| TM7 | M527 | 89.5 |
| TM7 | A528 | 44.4 |
| EL4 | R531 | 15.2 |
| EL4 | V533 | 34.2 |
| EL4 | I535 | 42.9 |
| EL4 | V538 | 56.5 |
| EL4 | P543 | 57.7 |
| EL4 | I545 | 34.5 |
| EL4 | A546 | 16.5 |
| EL4 | F547 | 58.3 |
| EL4 | V548 | 46.3 |
| EL4 | Y550 | 60.6 |
| EL4 | A553 | 39.0 |
| EL4 | L554 | 35.8 |
| EL4 | T555 | 17.0 |
| EL4 | R556 | 42.7 |
| EL4 | L557 | 44.0 |
| EL4 | P558 | 36.2 |
| EL4 | L559 | 46.9 |
| EL4 | S560 | 35.4 |
| TM8 | F562 | 26.7 |
| TM8 | W563 | 81.6 |
| TM8 | A564 | 22.2 |
| TM8 | F567 | 83.1 |
| TM8 | F568 | 30.0 |
| TM8 | L571 | 46.9 |
| TM10 | I626 | 15.5 |
| TM10 | Q630 | 58.6 |

^a^An interaction is defined as a minimum distance between beads in the residues to be < 6 Å.
