# Supplementary material for: Membrane cholesterol regulates inhibition and substrate transport by the glycine transporter, GlyT2
Source: Life Sci Alliance. 2023 Jan 23;6(4):e202201708. doi: 10.26508/lsa.202201708 (PMC9873984; doi:10.26508/lsa.202201708)
Supplement: Supplementary file 3 [file LSA-2022-01708_TableS3.docx]

**Table S3 - Percentage of the CG simulation time with CHOL interacting in the vicinity of the LAS in which residues are in contact with the cholesterol residues that bound near the bottom of the extracellular allosteric pocket of GlyT2 when OLLeu is bound in the extracellular allosteric pocket.**

Only interactions that occur for >15% of the total simulation time are reported.^a^

| Region | Residue | Occupancy |
| --- | --- | --- |
| TM1 | K197 | 37.6 |
| TM1 | I198 | 51.7 |
| TM1 | I201 | 58.2 |
| TM1 | L202 | 50.5 |
| TM1 | V205 | 57.9 |
| TM1 | N213 | 73.5 |
| TM1 | V214 | 28.5 |
| TM1 | P218 | 30.2 |
| TM5 | T427 | 57.4 |
| TM5 | Y430 | 95.1 |
| TM5 | V431 | 66.3 |
| TM5 | L433 | 74.3 |
| TM5 | V434 | 95.8 |
| TM5 | L437 | 57.4 |
| TM7 | T502 | 25.5 |
| TM7 | L503 | 42.6 |
| TM7 | I504 | 72.8 |
| TM7 | T508 | 81.2 |
| TM7 | A511 | 74.3 |
| TM7 | T512 | 87.6 |
| TM7 | F515 | 91.8 |
| TM7 | A516 | 36.1 |
| TM7 | V519 | 37.9 |
| TM8 | F567 | 18.6 |
| TM8 | M570 | 34.7 |
| – | OLLeu | 29.5 |

^a^An interaction is defined as a minimum distance between beads in the residues to be < 6 Å.
