# Supplementary material for: Membrane cholesterol regulates inhibition and substrate transport by the glycine transporter, GlyT2
Source: Life Sci Alliance. 2023 Jan 23;6(4):e202201708. doi: 10.26508/lsa.202201708 (PMC9873984; doi:10.26508/lsa.202201708)
Supplement: Supplementary file 4 [file LSA-2022-01708_TableS4.docx]

**Table S4 - Percentage of the total simulation time in which residues are in contact with the lipid inhibitor bound in the LAS in atomistic simulations of the backmapped structure.**

Only interactions that occur for >15% of the total simulation time are reported.^a^

| Region | Residue | OLLeu | OLLys | OL-CARN | OLTrp |
| --- | --- | --- | --- | --- | --- |
| TM1 | N213 | 16.3 | 33.3 | - | - |
| TM1 | V214 | 23.9 | 54.7 | 48.6 | 43.9 |
| TM1 | W215 | 29.8 | - | - | - |
| TM1 | F217 | 19.6 | 67.6 | 54.8 | 40.6 |
| TM1 | P218 | 64.6 | 50.7 | 62.3 | 68.0 |
| TM5 | Y430 | 25.8 | 50.5 | 44.9 | 42.7 |
| TM5 | L433 | 26.1 | 42.8 | 24.2 | 40.1 |
| TM5 | V434 | - | 25.3 | 17.8 | 17.0 |
| TM5 | L437 | 50.9 | 77.8 | 53.7 | 74.8 |
| TM5 | G440 | 32.8 | 44.1 | 51.6 | 63.0 |
| TM5 | V441 | 33.2 | - | - | 15.7 |
| TM5 | T442 | 20.4 | - | - | 26.4 |
| TM7 | T512 | 20.1 | 41.9 | 29.3 | 33.0 |
| TM7 | F515 | 22.4 | 43.5 | 58.3 | 66.8 |
| TM7 | A516 | 34.8 | 62.5 | 51.1 | 50.9 |
| TM7 | V519 | 21.2 | 43.1 | 28.2 | 31.3 |
| TM7 | I520 | 57.9 | 66.5 | 60.3 | 71.5 |
| TM7 | V523 | 66.6 | 67.6 | 45.1 | 70.1 |
| TM7 | I524 | 17.4 | - | - | - |
| TM7 | F526 | 36.2 | 37.2 | 39.4 | 56.1 |
| TM7 | M527 | 69.3 | 61.1 | 53.7 | 78.8 |
| EL4 | N529 | - | 20.3 | - | - |
| EL4 | V533 | 19.7 | 48.7 | 75.4 | 47.5 |
| EL4 | I535 | 37.6 | 41.7 | 52.4 | 63.1 |
| EL4 | N537 | 32.0 | 51.5 | 35.9 | 67.5 |
| EL4 | V538 | 42.7 | 19.3 | 38.2 | 60.7 |
| EL4 | G542 | 25.3 | - | - | - |
| EL4 | P543 | 32.3 | - | - | - |
| EL4 | R556 | 26.9 | - | - | - |
| EL4 | P558 | 15.9 | - | - | - |
| EL4 | L559 | 27.3 | 18.5 | - | 61.4 |
| EL4 | S560 | 42.1 | 46.0 | 64.1 | 73.8 |
| TM8 | F562 | 69.2 | 98.3 | 97.4 | 90.5 |
| TM8 | W563 | 100.0 | 94.9 | 88.3 | 97.5 |
| TM8 | I566 | 52.7 | 68.6 | 68.4 | 65.7 |
| TM8 | F567 | 72.8 | 17.7 | 75.6 | 69.8 |
| TM8 | M570 | 66.5 | 73.6 | 74.9 | 69.4 |
| TM8 | L574 | - | 19.3 | - | - |
| TM10 | F629 | - | - | 21.4 | - |
| TM10 | N630 | 22.7 | - | - | - |

^a^An interaction is defined as a minimum distance between heavy atoms in the residues to be < 4 Å
