# Supplementary material for: Membrane cholesterol regulates inhibition and substrate transport by the glycine transporter, GlyT2
Source: Life Sci Alliance. 2023 Jan 23;6(4):e202201708. doi: 10.26508/lsa.202201708 (PMC9873984; doi:10.26508/lsa.202201708)
Supplement: Supplementary file 5 [file LSA-2022-01708_TableS5.docx]

**Table S5 - Percentage of the total simulation time (in atomistic simulations) in which residues are in contact with the cholesterol molecule bound near the bottom of the LAS of GlyT2 while a lipid inhibitor is bound in the extracellular allosteric pocket.**

Only interactions that occur for >30% of the total simulation time are reported.^a^

| Region | Residue | OLLeu | OLLys | OLCarn | OLTrp |
| --- | --- | --- | --- | --- | --- |
| TM1 | V205 | 37.7 | 52.9 | 60.7 | 55.1 |
| TM1 | G206 | - | - | 35.9 | 35.4 |
| TM1 | A208 | - | 42.5 | 47.3 | 37.5 |
| TM5 | Y430 | 65.3 | 54.5 | 70.4 | 56.4 |
| TM5 | V434 | - | 38.3 | 44.7 | 39.2 |
| TM7 | C507 | 34.3 | - | - | - |
| TM7 | T508 | 32.3 | 32.6 | - | - |
| TM7 | A511 | 43.1 | 31.1 | 43.8 | 38.3 |
| TM7 | T512 | 44.1 | 48.6 | 47.5 | 37.4 |
| TM7 | F515 | 56.3 | 55.7 | 66.1 | 72.6 |
| - | Membrane CHOL | 52.7 | - | - | - |

^a^An interaction is defined as a minimum distance between heavy atoms in the residues to be < 4 Å
