# Supplementary material for: Membrane cholesterol regulates inhibition and substrate transport by the glycine transporter, GlyT2
Source: Life Sci Alliance. 2023 Jan 23;6(4):e202201708. doi: 10.26508/lsa.202201708 (PMC9873984; doi:10.26508/lsa.202201708)
Supplement: Supplementary file 6 [file LSA-2022-01708_TableS6.docx]

**Table S6 - Membrane cholesterol depletion alters GlyT2 functionality^†^.**

| **Treatment** | **Kinetic Parameters** | |
| --- | --- | --- |
| **MβCD** | **K_m_ (µM)** | **V_max_** |
| Baseline | 18.59  (16.44 – 21.02) | 0.9984  (0.9666 – 1.031) |
| 0 mM | 15.26  (11.23 – 20.63) | 1.041  (0.9639 – 1.124) |
| 15 mM | 11.66  (8.57 – 15.78) | 0.5936****  (0.5508 – 0.6390) |
| **γCD** | K_m_ (µM) | V_max_ |
| **Baseline** | 27.02  (24.09 – 30.30) | 0.99  (0.96 – 1.03) |
| **15 mM** | 23.53  (18.19 – 30.41) | 0.86^**^  (0.80 – 0.92) |

**^†^** GlyT2 function was assessed by measuring transport dependent currents following application of increasing glycine concentrations (1-300 µM) before, and after, incubation of *Xenopus laevis* oocytes with methyl-β-cyclodextrin (MβCD) or γ-cyclodextrin (γCD) for 30 minutes at 32°C. Values are presented as mean (95% confidence interval) with n ≥ 5. Differences in K_m_ and V_max_ values between control and MβCD or γCD treated oocytes were determined using a two-way paired t-test.

Data information: Statistical significance is presented as * p ≤ 0.05, ** p ≤ 0.01, *** p ≤ 0.001 and *** p ≤ 0.0001.
