# Supplementary material for: Membrane cholesterol regulates inhibition and substrate transport by the glycine transporter, GlyT2
Source: Life Sci Alliance. 2023 Jan 23;6(4):e202201708. doi: 10.26508/lsa.202201708 (PMC9873984; doi:10.26508/lsa.202201708)
Supplement: Supplementary file 7 [file LSA-2022-01708_TableS7.docx]

**Table S7 - Activity of bioactive lipids on WT GlyT2 expressed in control and MβCD treated *Xenopus laevis* oocytes^†^.**

|  | **Condition** | | | |
| --- | --- | --- | --- | --- |
|  | **Control** | | **MβCD** | |
| **Compound** | **IC_50_ (nM)** | **Inhibition at 3 µM (%)** | **IC_50_ (nM)** | **Inhibition at 3 µM (%)** |
| **Oleoyl-L-Lysine** | 116  (91 – 147) | 93.6 ± 1.9 | 269^*^  (180 – 406) | 91.5 ± 1.5 |
| **Oleoyl-L-Carnitine** | 520^a^ | 55.5 ± 5.2^b^ | 800^a^ | 60.5 ± 5.3^b^ |
| **Oleoyl-L-Leucine** | 262  (195 – 352) | 69.4 ± 3.8 | 619^***^  (464 – 834) | 64.9 ± 3.8 |
| **Oleoyl-L-Tryptophan** | 103  (75 – 142) | 98.6 ± 0.3 | 130  (97 – 175) | 78 ± 4 |

**^†^** Activity of bioactive lipids was tested by examining the inhibition of currents produced by application of the glycine EC_50_ to WT GlyT2 expressed in *Xenopus laevis* oocytes. Cholesterol depletion was performed by incubating oocytes in 15 mM MβCD for 30 minutes at 32 °C. Values are presented as mean (95% confidence interval) or mean ± SEM (n ≥ 5). Differences between IC_50_ and inhibition at 3 µM values between control and treated cells were determined via two-tailed unpaired t-tests. Statistical significance is presented as * p ≤ 0.05, ** p ≤ 0.01, *** p ≤ 0.001 and *** p ≤ 0.0001.

^a^ 95% confidence intervals were unable to be reliably fit.

^b^ Values are representative of inhibition at 1 µM due to the chaotropic effects observed with exposure to 3 µM Oleoyl-L-Carnitine.
