# Supplementary material for: Membrane cholesterol regulates inhibition and substrate transport by the glycine transporter, GlyT2
Source: Life Sci Alliance. 2023 Jan 23;6(4):e202201708. doi: 10.26508/lsa.202201708 (PMC9873984; doi:10.26508/lsa.202201708)
Supplement: Supplementary file 8 [file LSA-2022-01708_TableS8.docx]

**Table S8 - Reversibility of bioactive lipid inhibition of WT GlyT2 expressed in *Xenopus laevis* oocytes pre and post cholesterol depletion^†^.**

|  | **Condition** | | | |
| --- | --- | --- | --- | --- |
|  | **Control** | | **MβCD** | |
| **Compound** | **Half-life**  **(min)** | **Recovery at**  **30 min (%)** | **Half-life**  **(min)** | **Recovery at**  **30 min (%)** |
| Oleoyl-L-Lysine | n.d.^a^ | 55.1 ± 1.5 | n.d.^a^ | 63.0 ± 4.1 |
| Oleoyl-L-Carnitine | n.d.^a^ | 45.7 ± 3.4 | n.d.^a^ | 92.4 ± 3.1^****^ |
| Oleoyl-L-Leucine | 2.3 ± 0.5 | 51.1 ± 2.7 | 3.1 ± 0.3 | 92.2 ± 3.7^****^ |
| Oleoyl-L-Tryptophan | No Recovery | No Recovery | No Recovery | No Recovery |

^†^ Reversibility of inhibitors was determined by co-applying an IC_50_ concentration of inhibitor with an EC_50_ concentration of glycine to *Xenopus laevis* oocytes expressing WT GlyT2 for 4 minutes. Following exposure to inhibitors, the EC_50_ of glycine was reapplied at 5-minute intervals for 30-minutes. Cholesterol depletion was performed by incubating oocytes in 15 mM MβCD for 30 minutes at 32°C. Oocytes were washed in recording buffer for 10 minutes after treatment to ensure the removal of residual MβCD. Values are presented as mean ± SEM with n ≥ 5 from at least two batches of oocytes. Differences in half-life and recovery at 30 min values between control and MβCD conditions were determined via two-tailed unpaired t-tests. Statistical significance is presented as * p ≤ 0.05, ** p ≤ 0.01, *** p ≤ 0.001 and **** p ≤ 0.0001.

^a^ Half-life was not determined as recovery did not plateau within the time course of the assay.
