# Supplementary material for: Membrane cholesterol regulates inhibition and substrate transport by the glycine transporter, GlyT2
Source: Life Sci Alliance. 2023 Jan 23;6(4):e202201708. doi: 10.26508/lsa.202201708 (PMC9873984; doi:10.26508/lsa.202201708)
Supplement: Supplementary file 9 [file LSA-2022-01708_TableS9.docx]

**Table S9 - EC_50_ values for glycine transport of WT GlyT2 and CHOL1 site mutant transporters expressed in *Xenopus laevis* oocytes^†^.**

| **Region** | **Mutant** | **Glycine EC_50_ (μM)** |
| --- | --- | --- |
| **WT** | - | 18  (16 – 21) |
| **TM1** | L198A | 28^**^  (25 – 31) |
| **TM5** | Y430L | 19  (17 – 22) |
|  | Y430F | 12  (10 – 13) |
| **TM7** | T512A | 90^****^  (80.76 – 99.83) |
|  | F515V | 7.8^*^  (6.7 – 9.1) |
|  | F515W | 7.6^**^  (6.5 – 8.8) |

^†^ Glycine EC_50_ values were determined by measuring glycine-dependent currents following application of increasing concentrations of glycine (1 μM – 3 mM) to *Xenopus laevis* oocytes expressing WT and mutant GlyT2 transporters. Currents were fit to the modified Michaelis-Menten equation, and raw currents were normalised to the V_max_ for each cell. Data is presented as mean (95% confidence interval) with n ≥ 3 from at least two batches of oocytes. A one-way ANOVA determined any differences in mutant glycine EC_50_ values compared to WT. Statistical significance is presented as * p ≤ 0.05, ** p ≤ 0.01, *** p ≤ 0.001 and **** p ≤ 0.0001.
