# Supplementary material for: Membrane cholesterol regulates inhibition and substrate transport by the glycine transporter, GlyT2
Source: Life Sci Alliance. 2023 Jan 23;6(4):e202201708. doi: 10.26508/lsa.202201708 (PMC9873984; doi:10.26508/lsa.202201708)
Supplement: Supplementary file 10 [file LSA-2022-01708_TableS10.docx]

Table S10 - Inhibitory activity of bioactive lipids at WT and CHOL1 mutant GlyT2 transporters expressed in *Xenopus laevis oocytes*^†^.

| **Mutation** | **Compound** | **IC_50_ (nM)** | **Inhibition at 3 μM (%)** |
| --- | --- | --- | --- |
| **Wild-Type** | Oleoyl-L-Lysine | 215  (136 – 340) | 86.6 ± 1.9 |
|  | Oleoyl-L-Carnitine | 520^a^ | 55.5 ± 5.2^b^ |
|  | Oleoyl-L-Leucine | 262  (195 – 352) | 69.4 ± 3.8 |
|  | Oleoyl-L-Tryptophan | 103  (75 – 142) | 76.2 ± 2.3 |
| **F515W** | Oleoyl-L-Lysine | 689  (299 – 1.78 μM) | 85.3 ± 6.4 |
|  | Oleoyl-L-Carnitine | 6.2 μM^a^ | 57.6 ± 2.3^b^ |
|  | Oleoyl-L-Leucine | 400  (307 – 525) | 86.7 ± 1.7^**^ |
|  | Oleoyl-L-Tryptophan | 128  (64 – 253) | 82.0 ± 3.9 |
| **Y430L** | Oleoyl-L-Lysine | 863^*^  (559 – 1.39 μM) | 69.7 ± 4.7^*^ |
|  | Oleoyl-L-Carnitine | 1.1 μM^a^ | 49.6 ± 2.9^b^ |
|  | Oleoyl-L-Leucine | 375  (223 – 642) | 63.8 ± 2.6 |
|  | Oleoyl-L-Tryptophan | 57  (39 – 85) | 83.5 ± 3.2 |
| **Y430F** | Oleoyl-L-Lysine | 123  (92 – 163) | 92.9 ± 1.7 |
|  | Oleoyl-L-Carnitine | 190^a^ | 71.0 ± 3.3^*b^ |
|  | Oleoyl-L-Leucine | 203  (129 – 316) | 76.7 ± 2.8 |
|  | Oleoyl-L-Tryptophan | 125  (91 – 171) | 84.5 ± 3.6 |
| **T512A** | Oleoyl-L-Lysine | 679  (413 – 1.16 μM) | 68.0 ± 1.7^**^ |
|  | Oleoyl-L-Carnitine | 1.7 μM^a^ | 44.1 ± 2.8^b^ |
|  | C18-L-L-leucine | 549  (300 – 1 μM) | 50.7 ± 5.7^**^ |
|  | Oleoyl-L-Tryptophan | 85  (62 – 116) | 74.4 ± 4.5 |

^†^ Inhibitory activity of bioactive lipids and ORG25543 was tested by examining the inhibition of glycine currents induced by application of the glycine EC_50_ to WT and mutant GlyT2 expressed in *Xenopus laevis* oocytes. Values are presented as mean (95% confidence interval) or mean ± SEM with n ≥ 5 from at least two batches of oocytes. Differences in IC_50_ and level of inhibition between WT and mutant GlyT2 transporters were determined by a one-way ANOVA with a Tukey’s posthoc test. Statistical significance is presented as * p ≤ 0.05, ** p ≤ 0.01, *** p ≤ 0.001 and **** p ≤ 0.0001.

^a^ 95% confidence intervals were unable to be reliably fit.

^b^ Values are representative of inhibition at 1 µM due to the chaotropic effects observed with exposure to 3 µM Oleyol-L-Carnitine.
