# Supplementary material for: Membrane cholesterol regulates inhibition and substrate transport by the glycine transporter, GlyT2
Source: Life Sci Alliance. 2023 Jan 23;6(4):e202201708. doi: 10.26508/lsa.202201708 (PMC9873984; doi:10.26508/lsa.202201708)
Supplement: Supplementary file 11 [file LSA-2022-01708_TableS11.docx]

Table S11 - Reversibility of bioactive lipid inhibition of WT and CHOL1 mutant GlyT2 transporters expressed in *Xenopus laevis* oocytes^†^_._

| **Mutation** | **Compound** | **Half-life**  **(min)** | **Recovery at 30 Min (%)** |
| --- | --- | --- | --- |
| **Wild-Type** | Oleoyl-L-Carnitine | n.d.^a^ | 45.7 ± 3.4 |
|  | Oleoyl-L-Lysine | n.d.^a^ | 55.1 ± 1.5 |
|  | Oleoyl-L-Leucine | 2.3 ± 0.5 | 51.1 ± 2.7 |
|  | Oleoyl-L-Tryptophan | No Recovery | No Recovery |
| **F515W** | Oleoyl-L-Carnitine | n.d.^a^ | 60.1 ± 2.7^*^ |
|  | Oleoyl-L-Lysine | n.d.^a^ | 57.8 ± 4.1 |
|  | Oleoyl-L-Leucine | 2.0 ± 0.2 | 70.1 ± 1.9^***^ |
|  | Oleoyl-L-Tryptophan | No Recovery | No Recovery |
| **Y430L** | Oleoyl-L-Carnitine | n.d.^a^ | 80.6 ± 3.6^****^ |
|  | Oleoyl-L-Lysine | n.d.^a^ | 72.5 ± 2.1^***^ |
|  | Oleoyl-L-Leucine | n.d.^b^ | 67.9 ± 2.3^***^ |
|  | Oleoyl-L-Tryptophan | No Recovery | No Recovery |
| **Y430F** | Oleoyl-L-Carnitine | n.d.^a^ | 24.7 ± 3.6^**^ |
|  | Oleoyl-L-Lysine | n.d.^a^ | 68.5 ± 2.1^**^ |
|  | Oleoyl-L-Leucine | n.d.^a^ | 26.6 ± 2.3^****^ |
|  | Oleoyl-L-Tryptophan | No Recovery | No Recovery |
| **T512A** | Oleoyl-L-Carnitine | n.d.^a^ | 79.4 ± 1.4^****^ |
|  | Oleoyl-L-Lysine | n.d.^a^ | 63.5 ± 2.0 |
|  | Oleoyl-L-Leucine | 1.7 ± 0.3 | 76.2 ± 4.1^****^ |
|  | Oleoyl-L-Tryptophan | No Recovery | No Recovery |

^†^ Reversibility of inhibitors was determined by co-applying an IC_50_ concentration of inhibitor with an EC_50_ concentration of glycine to *Xenopus* *laevis* oocytes expressing WT and mutant GlyT2 transporters for 4 minutes. Following exposure to inhibitors, the EC_50_ of glycine was reapplied at 5-minute intervals for 30-minutes. Values are presented as mean ± SEM with n ≥ 5 from at least two batches of oocytes. Differences in half-life and recovery after 30-minutes between WT and mutant GlyT2 transporters were determined via a one-way ANOVA with a Tukey’s posthoc test. Statistical significance is presented as * p ≤ 0.05, ** p ≤ 0.01, *** p ≤ 0.001 and **** p ≤ 0.0001.

^a^ Half-life was not determined as recovery did not plateau within the time course of the assay. ^b^ Half-life was not determined as the data could not be reliably fit.
