# Supplementary material for: Membrane cholesterol regulates inhibition and substrate transport by the glycine transporter, GlyT2
Source: Life Sci Alliance. 2023 Jan 23;6(4):e202201708. doi: 10.26508/lsa.202201708 (PMC9873984; doi:10.26508/lsa.202201708)
Supplement: Supplementary file 12 [file LSA-2022-01708_TableS12.docx]

**Table S12 - Percentage of the total simulation time in which residues are in contact with Oleoyl-L-Carnitine bound in the LAS in atomistic simulations of GlyT2 mutants.^a^**

| Region | Residue | WT | T512A | Y430F | Y430L | F515W |
| --- | --- | --- | --- | --- | --- | --- |
| TM1 | V214 | 48.6 | 56.1 | 42.7 | 57.6 | 73.6 |
| TM1 | W215 | - | - | - | - | 33.7 |
| TM1 | F217 | 54.8 | 69.0 | 47.3 | 52.1 | 64.5 |
| TM1 | P218 | 62.3 | 46.2 | 68.3 | 67.6 | 60.5 |
| TM5 | Y430 | 44.9 | 19.2 | - | - | 17.3 |
| TM5 | L433 | 24.2 | 35.8 | 33.6 | 45.7 | 16.6 |
| TM5 | V434 | 17.8 | - | - | - | - |
| TM5 | L437 | 53.7 | 60.5 | 55.3 | 25.8 | 34.4 |
| TM5 | R439 | - | - | 89.2 | 82.7 | 81.4 |
| TM5 | G440 | 51.6 | 41.3 | 88.0 | 76.1 | 90.8 |
| TM5 | V441 | - | 15.5 | 44.7 | 18.8 | 25.4 |
| TM5 | T442 | - | 20.2 | 79.8 | 47.9 | 58.3 |
| TM7 | T512 | 29.3 | 31.6 | 29.8 | 24.2 | - |
| TM7 | F515 | 58.3 | 22.8 | - | 39.0 | 22.4 |
| TM7 | A516 | 51.1 | 56.6 | 52.5 | 60.7 | 42.3 |
| TM7 | V519 | 28.2 | 45.9 | 46.4 | 42.3 | 27.9 |
| TM7 | I520 | 60.3 | 69.8 | 75.6 | 77.0 | 72.7 |
| TM7 | V523 | 45.1 | 59.7 | 72.8 | 61.1 | 75.7 |
| TM7 | F526 | 39.4 | 33.7 | 57.6 | 41.1 | 55.5 |
| TM7 | M527 | 53.7 | 59.8 | 40.6 | 46.9 | 58.5 |
| EL4 | V533 | 75.4 | 51.7 | 40.1 | 38.4 | 56.1 |
| EL4 | I535 | 52.4 | 68.4 | 24.6 | 35.2 | 16.1 |
| EL4 | N537 | 35.9 | 75.9 | - | 37.0 | - |
| EL4 | V538 | 38.2 | 32.9 | - | 23.7 | - |
| EL4 | L559 | - | 18.3 | - | - | - |
| EL4 | S560 | 64.1 | 63.5 | 22.6 | 62.4 | 75.6 |
| TM8 | F562 | 97.4 | 93.7 | 98.9 | 99.2 | 98.8 |
| TM8 | W563 | 88.3 | 90.2 | 96.4 | 96.7 | 98.4 |
| TM8 | I566 | 68.4 | 71.1 | 70.7 | 67.1 | 67.6 |
| TM8 | F567 | 75.6 | 55.5 | 65.9 | 64.5 | 88.8 |
| TM8 | M570 | 74.9 | 58.0 | 71.9 | 51.7 | 78.7 |
| TM8 | L571 | - | - | - | - | 27.3 |
| TM8 | T573 | - | - | 33.5 | 33.2 | - |
| TM10 | F629 | 21.4 | - | - | - | 43.5 |
| ^a^ Only interactions that occur for >15% of the total simulation time are reported. An interaction is defined as a minimum distance between heavy atoms in the residues to be < 4 Å. | | | | | | |
