# Supplementary material for: Membrane cholesterol regulates inhibition and substrate transport by the glycine transporter, GlyT2
Source: Life Sci Alliance. 2023 Jan 23;6(4):e202201708. doi: 10.26508/lsa.202201708 (PMC9873984; doi:10.26508/lsa.202201708)
Supplement: Supplementary file 13 [file LSA-2022-01708_TableS13.docx]

**Table S13 -**  Percentage of the total simulation time in which residues are in contact with the cholesterol molecule bound near the bottom of the LAS of GlyT2 while Oleoyl-L-Carnitine is bound in the extracellular allosteric pocket. Only interactions that occur for >30% of the total simulation time are reported.^a^

| Region | Residue | WT | T512A | Y430F | Y430L | | F515W | |
| --- | --- | --- | --- | --- | --- | --- | --- | --- |
| TM1 | V205 | 60.7 | 69.5 | 94.7 | 84.6 | | 62.7 | |
| TM1 | G206 | 35.9 | - | 80.9 | 82.7 | | - | |
| TM1 | Y207 | - | - | - | 65.3 | | - | |
| TM1 | A208 | 47.3 | 66.1 | 70.3 | 53.9 | | 26.6 | |
| TM5 | Y430 | 70.4 | 98.5 | 98.2 | 63.4 | | 71.4 | |
| TM5 | L433 | - | - | 50.1 | 60.5 | | - | |
| TM5 | V434 | 44.7 | 62.7 | 61.1 | 53.5 | | 57.3 | |
| TM5 | L437 | - | 32.6 | 38.9 | 53.9 | | - | |
| TM7 | T508 |  | 56.0 | 39.6 | - | | - | |
| TM7 | A511 | 43.8 | 49.7 | 63.5 | 37.6 | | 36.0 | |
| TM7 | T512 | 47.5 | 50.8 | 77.2 | 60.5 | | 58.5 | |
| TM7 | F515 | 66.1 | 88.0 | 93.1 | 94.5 | | 86.6 | |
| TM8 | T573 | - | - | 62.7 | 54.8 | | - | |
| ^a^An interaction is defined as a minimum distance between heavy atoms in the residues to be < 4 Å. | | | | | |  | |  |
